# Supplementary material for: PDKit: A data science toolkit for the digital assessment of Parkinson’s Disease
Source: PLoS Comput Biol. 2021 Mar 12;17(3):e1008833. doi: 10.1371/journal.pcbi.1008833 (PMC7990207; doi:10.1371/journal.pcbi.1008833)
Supplement: Attachment — Submitted filename: responsetoreviewers.pdf [file pcbi.1008833.s002.pdf]

Response To Reviewers

We would like to thank reviewers for their comments on our manuscript. We address each reviewer's comments separately. The reviewer's comments are italicised.

Reviewer 1

- *The paper presents PDKit, a software tool aimed at collecting and analysing data related to Parkinsons Disease and derived from wearables and behavioural monitoring. The last step of the tool is to provide a prediction of the severity of the disease with respect to standard clinical evaluations. The paper can be classified as a technical paper, describing the main software modules composing the tool and the general features of each module, presenting only some simple application examples. The presented results derive from previous studies on big datasets collected during experimental campaigns. These results have been already published by the authors in dedicated papers. The main novelty of the paper is the presentation of the software architecture of the tool, which is also available to researchers and it has been already dowloaded several times.*

  We thank the reviewer for the precise description of the main contribution of this paper. We moreover confirm that the paper has been submitted for consideration for the special section of the journal targeting specifically the publication of software made available under an Open Source Initiative (OSI) compliant license which "has been shown to provide new biological insights, either as a part of the software article, or published elsewhere" rather than as a standard research paper.

- *However, the description of the tool lacks of specific examples of its applications, for example referring to the experimental results presented in Section Results. The authors claim that they provide specific examples and use cases in notebooks, available for download, and in the Read-the-Docs files, but not describing in detail this part in the paper, it reduces the novel contribution of the paper.*

  We fully acknowledge that the initial submission of this work relied on external documents available on github and RTD for examples. To address this, we have included a new subsection entitled "CUSSP Data Analysis using PDKit" in the Results section which provides a more detailed description of the use of PDKit that includes a wider commentary.

- *In addition, a more detailed description of the advantages of using the tool, for example from the medical users (in terms of usability and acceptance) would provide an important added-value for the paper. For all these reasons, I suggest the authors to review the paper including these missing parts.*
  We have added additional material in Section 1 to specifically identify the advantages offered by the toolkit to medical users. With regards to acceptance and usability we have updated the information related to the adoption of the toolkit as indicated by quantitative and qualitative metrics in the paragraph "Implementation and Release"; highlight the explicit links of software implementation to published research literature in the new section "CUSSP Data Analysis using PDKit" and further demonstrated in the description of its use in the subsection CUSSP Data Analysis using PDKit in the Results section.

Reviewer 2

- *This manuscript describes a tool kit developed by the authors with to facilitate the development and open sharing of novel digital biomarkers for PD and hence help address the current lack of algorithmic and model transparency . This goal is extremely worthwhile for PD and other diseases, where digital biomarkers and digital clinical outcome assessment are becoming increasingly widely used, often without well characterised performance or transparent algorithms. And the point is well made that this is a barrier to acceptance of these methods by regulators.*

  We also thank Reviewer 2 for the precise description of the main contribution of this paper. We moreover confirm that the paper has been submitted for consideration for the special section of the journal targeting specifically the publication of software made available under an Open Source Initiative (OSI) compliant license which "has been shown to provide new biological insights, either as a part of the software article, or published elsewhere" rather than as a standard research paper.

- *These results are from quite a large data volume, and I was surprised to see so little comment on them and no reference in the conclusions.*

  In view of the above point relating to this submission specifically for consideration for inclusion to the Software Section of this journal and its particular requirements for submission, a detailed discussion of the conclusions of the CUSSP study is beyond the scope of this paper. Instead, as per the guidelines for submissions in this section, we only include a brief summary of the key outcomes of that study and provide up to date references to open access papers which present the detailed analysis, results and our conclusions.

- *The content is very interesting, but I think readers may find it hard to follow the flow of the paper, as the results section that involves results obtained by processing data from CUSSP, seems out of place, without proper method or discussion of the results. These results are from quite a large data volume, and I was surprised to see so little comment on them and no reference in the conclusions. I would suggest a refinement of the structure to address this concern, including expanding these results, having a clear description of how the toolkit enabled this analysis, plus discussion ad reference in conclusions, so that readers can get an example of the application of the tookit to generate novel results.*

  We agree that a description of the process of conducting an analysis using PDkit should be mainly self-standing and to this end we have introduced a new sub-section entitled "CUSSP Data Analysis using PDKit" in the Results section which provides a more detailed description of the use of PDKit that includes a wider commentary.

  Furthermore, we have added the following statement in the conclusions
  'The use of PDKit in the analysis of CUSSP made it straightforward to perform not just the analysis based on pre-specified features that used a standard statistical classifier, but to also subsequently perform a very broad exploratory analysis over a large number of features and classifiers.'

More minor comments.

- *Line 139: One approach suggests that features employed for symptom assessment should reflect biomedical intuition based on clinical experience, with the opposing view exhorting the advantages of a purely data-driven approach. A patient rather than clinical experience perspective should also be mentioned her, see FDA patient focused drug development programmes.*

  We have replaced Line 139 with the following:

  One approach suggests that features employed for symptom assessment should reflect biomedical intuition based on clinical experience as well as what is important to patients, with the opposing view exhorting the advantages of a purely data-driven approach

- *Line 162. Given the focus on regulatory issues, please clarify some of the terminology, and in particular the difference between Biomarker and Clinical outcome assessments (see various references on the EMA and FDA web site https://www.fda.gov/drugs/development-approval-process-drugs/drug-development-tool-ddt-qualification-programs). The digital technology that are the focus of this paper might be either biomarkers or clinical outcome assessments, but more likely the latter.*

  Last line of the section Clinical scores we have included the sentence:
  'It is envisioned that PDKit can be used not just for the development of effective biomarkers but also for clinical outcome assessments in that there is potential to replace the MDS-UPDRS clinician-reported outcome.'
